# Intersectional Discrimination and Mental Health in Later Life: Ageism as a Core Dimension

**DOI:** 10.1093/geroni/igaf122.2205

**Published:** 2025-12-31

**Authors:** Yi Wang, Yifan Lou, Huei-wern Shen, Ernest Gonzales

**Affiliations:** University of Iowa School of Social Work, Iowa City, Iowa, United States; Virginia Commonwealth University, Richmond, Virginia, United States; University of North Texas, Denton, Texas, United States; New York University, New York, New York, United States

## Abstract

Despite extensive literature that examines the relationship between discrimination and health, less is known about specific discrimination attributions and how they may differentially affect health outcomes. To address this gap, the current study investigated the latent typology of the discrimination attributions, and the effects of intersectional attributions on mental health in later life. Data came from 6,282 respondents in the 2016 Psychosocial Leave-Behind Questionnaire of the Health and Retirement Study. Participants ascribed their everyday discrimination experiences to a list of ten potential reasons (e.g., ethnicity, ancestry, gender, race, age, religion, financial). Latent class analysis (LCA) was performed to identify discrimination attribution typologies. Regression models with marginal effects were conducted to explore differential mental health effects of different attribution typologies. Five distinct typologies were identified: few discrimination experiences (33%), discrimination with no specified attributes (5%), discrimination due to age (48%), discrimination due to age, race, ethnicity (8%), and discrimination due to age and explicit characteristics (5%) such as physical disability and appearance. Regression analysis revealed significant associations between discrimination and depressive symptoms, cognitive function, and loneliness. Discrimination involving more than just age, especially explicit characteristics, had stronger negative effects on mental health. Ageism emerged as a core dimension and prevalent theme across multiple discrimination experiences, and often co-occurs with other characteristics, highlighting the intersectionality of discrimination. The negative mental health effects were most pronounced for those who experienced discrimination related to intersectional attributions. Implications for practice and research were discussed.

